# Intraperitoneal Copper–Manganese Nanozyme Suppresses Peritoneal Metastasis with Antitumor Immune Activation

**DOI:** 10.34133/bmr.0401

**Published:** 2026-09-09

**Authors:** Jinquan Zhang, Zhenze Xie, Muye He, Siqi He, Ling Wang, Chengxiao Song, Jingguang Wang, Jian Li, Chang Du, Ruoxu Dou

**Affiliations:** ^1^ Department of Hepatobiliary Surgery, The Fifth Affiliated Hospital, Sun Yat-Sen University, Zhuhai, Guangdong 519000, PR China.; ^2^ Department of Biomedical Engineering, School of Materials Science and Engineering, South China University of Technology, Guangzhou, Guangdong 510641, PR China.; ^3^ National Engineering Research Center for Tissue Restoration and Reconstruction, South China University of Technology, Guangzhou, Guangdong 510006, PR China.; ^4^ Comprehensive Cancer Center, Foresea Life Insurance Guangzhou General Hospital, Guangzhou, Guangdong 511300, PR China.

## Abstract

Peritoneal metastasis from colorectal cancer (CRC) carries a dismal prognosis, and current surgical and medical therapies achieve limited efficacy with high recurrence rates. Here, we developed a copper–manganese-based nanozyme (CM) that integrated MnO_2_-mediated Fenton-like catalysis with copper-induced cuproptosis for intraperitoneal treatment of CRC peritoneal metastasis. Composed of CuO and Cu_2_(OH)_3_Cl crystalline domains embedded in amorphous MnO_2_, the nanozyme has a hydrodynamic diameter of ~140 nm and displays peroxidase-like, glutathione oxidase, catalase, and Fenton-like activities that amplify reactive oxygen species (ROS) under tumor-mimicking conditions. CRC cells efficiently internalize the nanozyme, which penetrates 3-dimensional spheroids and induces strong, selective cytotoxicity via ROS amplification and cuproptosis. Intraperitoneal delivery of CM achieves prolonged peritoneal retention, marked reduction of peritoneal tumor burden and ascites, and remodeling of the local immune microenvironment with activation of antitumor immune responses. This study provides an effective cavity-confined therapeutic approach for CRC peritoneal metastasis based on Fenton-like ROS amplification and cuproptosis.

## Introduction

Peritoneal metastasis from colorectal cancer (CRC) represents a highly lethal pattern of disease spread with very limited treatment options. It develops in approximately 8% to 13% of patients with CRC and, once established, is associated with a median overall survival of only about 16 months [1,2]. Current treatment strategies rely mainly on systemic chemotherapy and cytoreductive surgery (CRS) with or without hyperthermic intraperitoneal (IP) chemotherapy (HIPEC) in selected patients. For carefully chosen patients with limited peritoneal disease treated in high-volume centers, CRS combined with HIPEC can achieve median overall survival approaching 5 years [3]. In routine practice, however, only a small subset of patients meet the strict criteria for such extensive surgery, and even after apparently complete cytoreduction, peritoneal recurrence rates as high as 75% have been reported [4]. Moreover, adding HIPEC to CRS increases perioperative morbidity, while recent randomized data have failed to show a clear overall survival advantage of combining HIPEC to CRS [3]. The limited tissue penetration of IP chemotherapeutics, the presence of poorly perfused tumor nodules, and substantial local and systemic toxicity remain barriers to better outcomes. Together, these factors underscore an urgent need for effective and safe drug-based strategies that can be administered repeatedly and more efficiently debulk diffuse colorectal peritoneal metastases.

In this context, nanozymes have recently emerged as powerful tools for cancer therapy [5,6]. Transition-metal-oxide nanozymes can mimic oxidase, peroxidase, catalase, and glutathione oxidase activities, thereby remodeling redox homeostasis in the tumor microenvironment and amplifying reactive oxygen species (ROS) [7,8]. In particular, MnO_2_-containing nanozymes can decompose endogenous H_2_O_2_, deplete glutathione (GSH), and catalyze Fenton-like reactions to generate highly cytotoxic hydroxyl radicals, especially under the mildly acidic conditions typical of tumors [8–10]. However, drug delivery to peritoneal tumor nodules is often compromised by limited perfusion and dense stromal architecture, which restricts the depth and uniformity of exposure achieved by conventional chemotherapeutics. In contrast, transition-metal-oxide nanozymes can catalytically convert endogenous substrates (e.g., H_2_O_2_) while concurrently eroding antioxidant buffering (e.g., via GSH depletion), thereby producing sustained local oxidative stress that may better address poorly vascularized, disseminated peritoneal metastases.

Concurrently, the recently described cell death modality “cuproptosis” has opened new avenues for copper-based cancer therapy. Copper-dependent death is triggered by direct binding of Cu^+^ to lipoylated components of the tricarboxylic acid cycle, leading to aggregation of lipoylated proteins, loss of iron–sulfur cluster proteins, proteotoxic stress, and ultimately cell death [11]. A growing body of work has exploited cuproptosis for cancer therapy using copper-based nanomaterials, including CuH nanoparticles, copper metal–organic frameworks (Cu-MOFs), and ROS-responsive nanoparticles co-delivering copper and the copper ionophore elesclomol [12–14]. These systems demonstrate that combining copper-induced cuproptosis with ROS-mediated oxidative damage or chemodynamic therapy can produce potent antitumor effects and, in some cases, trigger favorable immune remodeling [15,16]. Nevertheless, most reported platforms have been evaluated in subcutaneous or orthotopic solid tumors, and their potential for treating peritoneal metastases from CRC has not been systematically investigated.

**Fig. 1.**
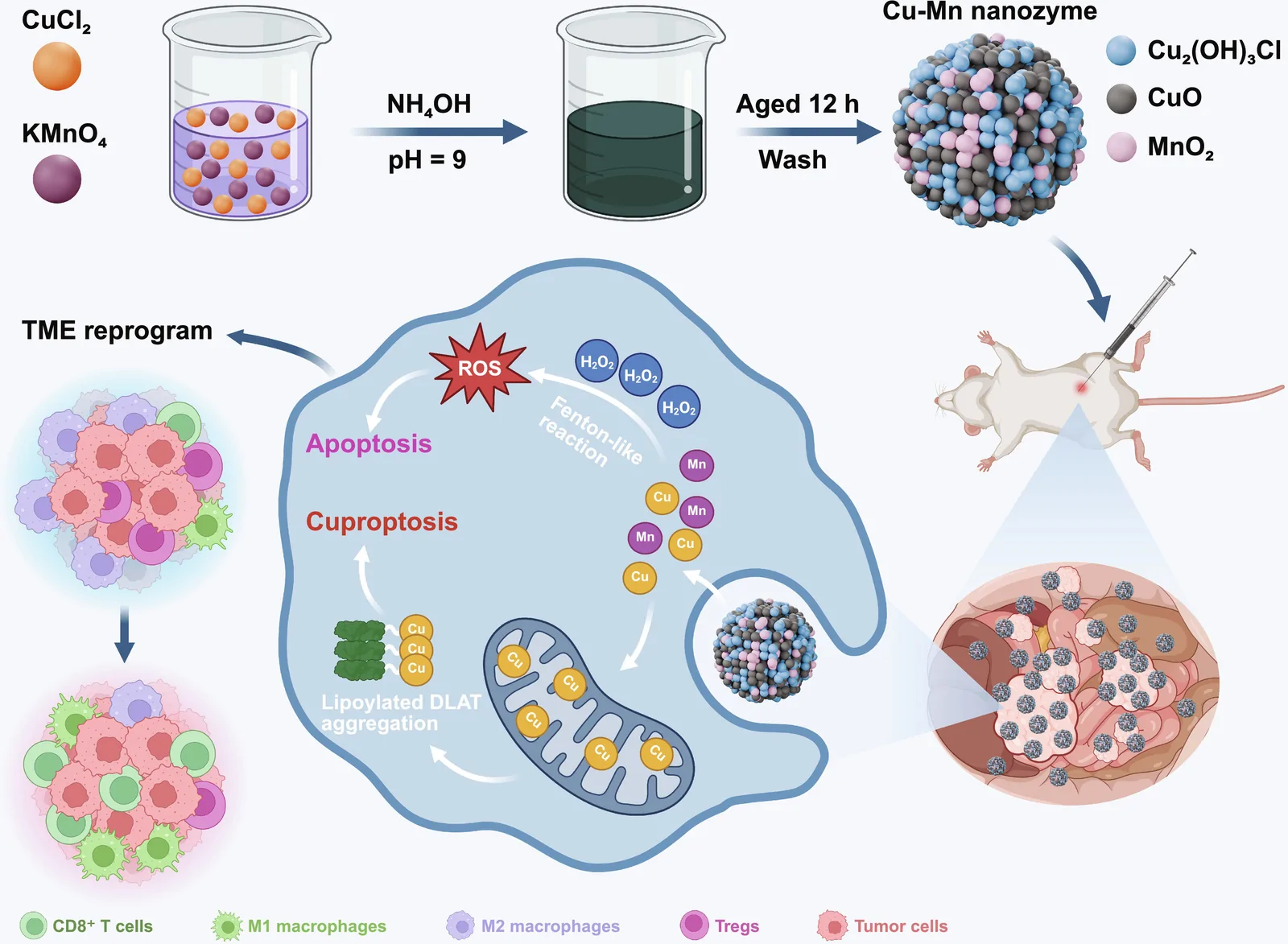
Schematic illustration of IP copper–manganese nanozyme therapy for peritoneal metastasis. CM is synthesized as an integrated nanoparticle assembled from subparticles, incorporating CuO/Cu_2_(OH)_3_Cl and amorphous MnO_2_. After IP administration, CM resides within the peritoneal cavity and reaches disseminated tumor nodules, entering tumor cells and penetrating compact 3D architectures. In H_2_O_2_- and GSH-containing tumor-relevant environments, CM drives multi-pathway redox modulation, including H_2_O_2_ processing, GSH consumption, O_2_ generation in catalase-like assays, and Fenton-like oxidative conversion under acidic conditions, thereby amplifying oxidative stress and triggering copper-associated mitochondrial dysfunction with DLAT aggregation and reduced ferredoxin 1 (FDX1), consistent with cuproptosis-associated proteotoxic stress. This catalytic stress translates into reduced peritoneal tumor burden and microenvironment remodeling, characterized by increased CD8^+^ T cell infiltration, decreased Foxp3^+^ regulatory T cells, and M1-skewed macrophage polarization.

Based on these considerations, we designed a copper–manganese-based nanozyme (abbreviated as CM) that integrated MnO_2_-mediated Fenton-like catalysis with Cu-driven cuproptosis into a single IP administered nanoplatform. CM consisted of CuO and Cu_2_(OH)_3_Cl crystalline domains embedded within an amorphous MnO_2_ matrix, forming a nanozyme with multiple catalytic activities and a hydrodynamic diameter of approximately 140 nm, which favored prolonged peritoneal retention while limiting systemic dissemination. By processing H_2_O_2_, oxidizing GSH, and promoting Fenton-like oxidative conversion under acidic conditions, the Mn-containing component was expected to aggravate redox stress in peritoneal tumor deposits and intracellular acidic compartments, whereas the Cu-containing component was expected to induce mitochondrial dysfunction and dihydrolipoamide S-acetyltransferase (DLAT) aggregation characteristic of cuproptosis. We hypothesized that IP delivery of CM would selectively accumulate on peritoneal surfaces, induce synergistic ROS-mediated damage and cuproptosis in CRC cells, and consequently shrink peritoneal metastases while promoting an immunostimulatory tumor microenvironment. The overall design and therapeutic mechanism of CM are illustrated in Fig. 1.

## Materials and Methods

### Synthesis of CM

The CM was prepared by chemical precipitation method. In brief, 0.02 M KMnO_4_ and 0.02 M CuCl_2_ were thoroughly mixed to form a solution, and ammonium hydroxide was used to adjust the pH solution to 9. Finally, the solution was aged for 12 h and washed 3 times to obtain CM.

### Physicochemical characterization

Transmission electron microscopy (TEM; FEI Talos-F200S, 200 kV) was used to observe the morphology and dispersity of CM. Low-magnification TEM images were acquired to evaluate the overall particle morphology and size uniformity across multiple nanoparticles, and high-resolution TEM (HRTEM) was used to examine lattice fringes within individual particles. Energy-dispersive x-ray spectroscopy (EDS) and x-ray photoelectron spectroscopy (XPS; Thermo Scientific Escalab Xi+) were used to analyze the elemental distribution and surface chemical states of CM. Powder x-ray diffraction (XRD) was performed to assess the crystalline characteristics of CM. Dynamic light scattering (DLS) was used to measure hydrodynamic size and polydispersity index (PDI) in normal aqueous dispersion and serum-containing solution. Zeta potential was determined by laser Doppler electrophoresis using a Zetasizer Nano ZS instrument (Malvern Instruments, UK).

For ion release analysis, CM was dispersed in buffers at pH 7.4 or pH 5.0. At the indicated time points, samples were collected and separated to obtain the supernatant, and the released Mn and Cu ions were quantified by inductively coupled plasma analysis. The release profiles were used to assess the pH-dependent metal-ion release behavior of CM.

### In vitro enzyme-mimetic activities

CM showed good enzyme-mimetic activities in tumor microenvironment, which contained high concentration of H_2_O_2_ and GSH. The peroxidase-like activity of CM was measured by the 2′,7′-dichlorodihydrofluorescein (DCFH) and H_2_O_2_. Briefly, 10 μM DCFH, 100 μM H_2_O_2_, and different concentrations of CM (0, 4, 20, 100, and 500 μg/ml) were added to phosphate-buffered saline (PBS) and incubated with gentle shaking for 20 min. Finally, the solution was measured using fluorescence spectrophotometer at λex = 488 nm, λem = 500 to 700 nm.

The glutathione oxidase activity of CM was evaluated using glutathione/5,5′-dithiobis(2-nitrobenzoic acid) (DTNB). Briefly, 1 mM GSH was mixed with different concentrations of CM (0, 4, 20, 100, and 500 μg/ml). After 30 min, 100 mM dimethyl sulfoxide (DMSO) solution of DTNB was introduced into solution and tested using ultraviolet–visible (UV–VIS) spectrophotometer at λ = 350 to 600 nm.

The catalase activity of CM was tested by measuring the O_2_ produced by decomposition of H_2_O_2_ in the presence of different concentrations of CM (0, 4, 20, and 100 μg/ml) through the dissolved oxygen meter (JPB-607A, INESA Scientific Instrument, Shanghai, China).

The Fenton-like reaction activity was determined by 3,3′,5,5′-tetramethylbenzidine (TMB) with H_2_O_2_. Briefly, 100 μM TMB, 100 μg/ml H_2_O_2_, and different concentrations of CM (0, 4, 20, 100, and 500 μg/ml) were added into PBS (pH = 5.0, 6.4, and 7.4), and the absorbance spectra were recorded from 300 to 800 nm using a UV–VIS spectrophotometer.

For enzyme kinetic analysis, H_2_O_2_ concentration was varied, while the CM concentration was kept constant. Initial reaction rates were calculated from the linear range of the reaction curves. Apparent Michaelis–Menten parameters were obtained by Michaelis–Menten fitting and Lineweaver–Burk analysis. The apparent kinetic parameters of CM were further compared with those of representative nanozymes reported in the literature.

### Cell culture

Murine colon carcinoma CT26 and CT26-luc cells, human gastric cancer MKN-45, human CRC DLD-1 and Caco2 cells, and murine fibroblasts L929 were kindly provided by R. Luo and W. Sun and were handled in accordance with the institutional biosafety guidelines of Sun Yat-Sen University. Cells were cultured in RPMI 1640 supplemented with 10% fetal bovine serum (FBS), 100 U/ml penicillin, and 100 μg/ml streptomycin at 37 °C in a humidified 5% CO_2_ incubator. Cells were passaged every 2 to 3 d and used within 15 passages.

### Cellular uptake and 3-dimensional spheroid penetration

For fluorescence-based tracking, CM was first modified with polyethyleneimine (PEI) and subsequently labeled with fluorescein isothiocyanate (FITC). Briefly, CM (10 mg) was dispersed in deionized water and mixed with PEI (10 mg) under gentle stirring at room temperature for 24 h. The PEI-modified nanozyme (CM-PEI) was collected by centrifugation and washed 3 times with deionized water to remove excess PEI. CM-PEI was then redispersed in carbonate buffer (pH 9.0) containing FITC (1 mg) and stirred at room temperature for 24 h in the dark. The resulting FITC-CM was harvested by centrifugation, washed repeatedly with PBS to remove unbound FITC, and finally redispersed in PBS for subsequent experiments.

For 2D uptake studies, CT26 cells were seeded in glass-bottom dishes at 1 × 10^5^ cells/well and allowed to adhere overnight. Cells were incubated with FITC-CM (15 μg/ml) for 1 to 7 h, washed 3 times with PBS, fixed with 4% paraformaldehyde (PFA) for 15 min, and counterstained with 4′,6-diamidino-2-phenylindole (DAPI). Images were acquired using an inverted fluorescence microscope with identical exposure settings. For quantitative analysis, fresh CT26 cells were seeded in 6-well plates and incubated with FITC-CM for 1 to 48 h, after which cells were harvested with trypsin, washed with PBS, and analyzed by flow cytometry in the FITC channel. Data were analyzed using FlowJo version 10.10.0.

Multicellular tumor spheroids were generated using ultra-low-attachment 96-well plates. CT26 cells (3 × 10^3^ per well) were cultured in complete medium for 3 d until compact spheroids formed. Spheroids were incubated with FITC-CM (15 μg/ml) for 12, 24, and 48 h, washed, and fixed with PFA. Z-stack images were acquired on a ZEISS LSM 980 confocal laser scanning microscope (CLSM) (λex = 488 nm), and a central optical section of each spheroid was selected to evaluate nanoparticle penetration. For quantitative evaluation, the spheroid region of interest (ROI) was outlined, and area-normalized FITC fluorescence intensity was measured using ImageJ. Spheroid diameters were measured from bright-field images to verify size consistency among groups.

### Cell viability and live/dead staining

Cytotoxicity was evaluated using a Cell Counting Kit-8 (CCK-8) assay. MKN-45, DLD-1, Caco2, CT26, and L929 cells were seeded in 96-well plates at 8 × 10^3^ cells per well and incubated overnight. Cells were treated with CM at concentrations from 0 to 100 μg/ml for 24 h. After incubation, culture medium was replaced with fresh medium containing 10% CCK-8 reagent, and plates were incubated for 1 h at 37 °C. Absorbance at 450 nm was measured, and cell viability was calculated relative to untreated controls. Half-maximal inhibitory concentration (IC₅₀) values were determined by nonlinear regression using GraphPad Prism version 9.

For live/dead staining, CT26 and L929 cells were treated with CM at 3.75, 7.5, 15, 30, and 60 μg/ml for 24 h. Cells were then incubated with calcein-AM (2 μM) and propidium iodide (PI; 8 μM) for 15 min at 37 °C in the dark, washed with PBS, and imaged by fluorescence microscopy.

### Assessment of mitochondrial membrane potential and intracellular ROS

Mitochondrial membrane potential (MMP) was measured using a JC-10 assay kit. CT26 cells were seeded in 12-well plates and treated with CM at 0, 15, 30, and 60 μg/ml for 24 h. After treatment, cells were incubated with JC-10 working solution at 37 °C for 30 min, washed with buffer, and imaged by CLSM to visualize red (aggregated) and green (monomeric) signals. For quantitative analysis, cells were harvested and analyzed by flow cytometry, and the ratio of red/green fluorescence was calculated. Data were analyzed using FlowJo version 10.10.0.

Intracellular ROS levels were assessed using DCFH-DA. CT26 cells were treated with CM as above, incubated with DCFH-DA (2 μM) in serum-free medium at 37 °C for 30 min, washed, and imaged by fluorescence microscopy. Mean fluorescence intensity within ROIs was quantified using ImageJ.

### Intracellular GSH assay

Intracellular GSH levels were measured to validate CM-induced antioxidant depletion in CT26 cells. CT26 cells were treated with CM at the indicated concentrations for 1, 6, 12, and 24 h. After treatment, cells were collected and processed according to the instructions of the GSH assay kit. The GSH content was calculated from the standard curve and normalized to the corresponding sample amount. Data were analyzed to determine the time- and concentration-dependent effects of CM on intracellular GSH depletion.

### Immunofluorescence and Western blotting for cuproptosis markers

To examine cuproptosis-related protein changes, CT26 cells were treated with CM at 0, 15, 30, and 60 μg/ml for 24 h. For immunofluorescence, cells cultured on coverslips were fixed with 4% PFA, permeabilized with 0.2% Triton X-100, and blocked with 5% bovine serum albumin (BSA) for 1 h. Samples were incubated with primary antibodies against DLAT overnight at 4 °C, followed by fluorescence-conjugated secondary antibodies for 1 h at room temperature. Nuclei were counterstained with DAPI, and images were acquired by CLSM. For quantitative analysis of DLAT aggregation, punctate DLAT-positive aggregates were segmented using ImageJ, and the aggregate area was normalized to cell number in each field.

For Western blotting, treated CT26 cells were lysed in Triton X-100-containing lysis buffer containing protease and phosphatase inhibitors on ice. After centrifugation at 4 °C, the supernatant was collected as the soluble fraction, while the pellet was washed and solubilized in sodium dodecyl sulfate (SDS)-containing loading buffer as the insoluble fraction. Equal amounts of protein (20 μg) were separated by SDS-PAGE (polyacrylamide gel electrophoresis) and transferred onto polyvinylidene difluoride (PVDF) membranes. Membranes were blocked with 5% non-fat milk and incubated with primary antibodies against DLAT, FDX1, and glyceraldehyde-3-phosphate dehydrogenase (GAPDH) (loading control) overnight at 4 °C, followed by horseradish peroxidase (HRP)-conjugated secondary antibodies for 1 h at room temperature. Bands were visualized using enhanced chemiluminescence and quantified using Image Lab version 6.1. Uncropped membrane images are provided in the Supplementary Materials.

### Animals and peritoneal metastasis model

All animal procedures were approved by the Institutional Animal Care and Use Committee of Sun Yat-sen University under approval no. SYSU-IACUC-2025-000581 and were performed in accordance with institutional and national guidelines for the care and use of laboratory animals. Female BALB/c mice (6 to 8 weeks old, 18 to 22 g) were purchased from Laboratory Animal Center of Sun Yat-Sen University (Guangzhou, China) and acclimatized for at least 1 week with free access to food and water under a 12-h light/dark cycle. To establish the peritoneal metastasis model, CT26-luc cells were harvested during logarithmic growth, washed, and resuspended in PBS. A suspension of 8 × 10^5^ cells in 200 μl of PBS was injected IP into each mouse. Tumor engraftment was confirmed by in vivo bioluminescence imaging on day 5 after inoculation following IP injection of d-luciferin (150 mg/kg).

### In vivo biodistribution and systemic safety

To determine the maximum tolerated dose of CM, healthy BALB/c mice were randomly assigned to receive a single IP injection of CM at 0, 2, 5, 10, 20, 40, or 60 mg/kg (*n* = 3 per group). Body weight and general condition were monitored daily for 7 d, and survival was recorded. At the end of observation or upon reaching humane endpoints, blood was collected via retro-orbital sinus or cardiac puncture under anesthesia. Serum levels of alanine aminotransferase (ALT), aspartate aminotransferase (AST), alkaline phosphatase (ALP), urea, creatinine (CREA), creatine kinase (CK), lactate dehydrogenase (LDH), and high-sensitivity C-reactive protein (hs-CRP) were measured using an automated biochemical analyzer (Hitachi 3100). Major organs (heart, liver, spleen, lung, and kidney) were harvested, fixed in 4% PFA, embedded in paraffin, sectioned, and stained with hematoxylin and eosin (H&E) for histopathological examination.

For biodistribution studies, CM was labeled with FITC as described above. Healthy BALB/c mice received a single IP injection of FITC-CM (20 mg/kg, 200 μl). At predetermined time points (1, 6, 12, 24, 48, 72, 96, and 120 h), mice were anesthetized and imaged using an in vivo imaging system (IVIS Spectrum, PerkinElmer) with excitation/emission filters suitable for FITC. ROIs encompassing the abdomen were quantified for total radiant efficiency. At selected time points (24, 48, 72, 96, and 120 h), mice were sacrificed and major organs (stomach, small and large intestine, heart, liver, spleen, etc.) were collected for ex vivo imaging.

In a separate cohort bearing peritoneal metastases, FITC-CM (20 mg/kg) was injected IP, and tumor nodules together with major organs were harvested at 24 and 48 h for ex vivo imaging to assess preferential accumulation on tumor implants.

### Antitumor efficacy study

For therapeutic evaluation, CT26-luc peritoneal metastasis-bearing mice were randomized into 3 groups (*n* = 5 per group): (a) PBS (negative control, 200 μl), (b) oxaliplatin (OX; 5 mg/kg in 200 μl), and (c) CM (20 mg/kg in 200 μl). Treatments were administered IP every 48 h starting on day 6 after tumor inoculation for a total of 4 doses. Tumor burden was monitored by serial bioluminescence imaging on days 5 (baseline), 9, and 13. After injection of d-luciferin (150 mg/kg IP), mice were imaged under identical exposure conditions, and total abdominal photon flux was quantified using Living Image software version 4.4.

Body weight was measured every 2 d. At the study endpoint (day 14), mice were euthanized. The peritoneal cavity was opened and photographed to document the distribution and size of metastatic nodules and ascites volume. Representative tumor implants and adjacent peritoneum were collected, fixed in PFA, embedded in paraffin, and sectioned for H&E and Ki-67 immunohistochemistry. For Ki-67 staining, sections were incubated with anti-Ki-67 antibody followed by HRP-conjugated secondary antibody and 3,3′-diaminobenzidine (DAB) development. The percentage of Ki-67-positive area within viable tumor regions was quantified using ImageJ.

### Immunofluorescence and flow cytometry of tumor-infiltrating immune cells

To evaluate immune remodeling, fresh tumor nodules from each treatment group were processed for immunofluorescence and flow cytometry. For immunofluorescence, paraffin sections were stained with antibodies against CD8 (cytotoxic T cells), CD4 (helper T cells), Foxp3 [regulatory T cells (Tregs)], CD86 (M1-like macrophages), and CD206 (M2-like macrophages). After incubation with appropriate fluorescent secondary antibodies, nuclei were counterstained with DAPI. Images were acquired by using an automated digital slide scanner, and positive areas for each marker were quantified using ImageJ to calculate densities and M1/M2 ratios.

For flow cytometry, peritoneal lavage fluid and tumor tissues were collected. Tumors were minced and digested with collagenase IV (1 mg/ml) and deoxyribonuclease (DNase) I (0.1 mg/ml) at 37 °C for 30 min, filtered through a 70-μm cell strainer, and washed. Single-cell suspensions were blocked with anti-CD16/32 antibody and stained with fluorophore-conjugated antibodies against CD3, CD4, CD8, and Foxp3 for T cell profiling. Data were acquired on cytometer and analyzed using FlowJo version 10.10.0. T cell subsets were gated as CD3^+^CD4^+^, CD3^+^CD8^+^, and CD3^+^CD4^+^Foxp3^+^ (Treg). The detailed gating strategy is provided in the Supplementary Materials.

### Statistical analysis

All quantitative data are presented as mean ± standard deviation (SD) unless otherwise stated. Statistical analyses were performed using GraphPad Prism version 9. For comparisons between 2 independent groups, unpaired 2-tailed Student’s *t* test was used when variance homogeneity was satisfied; otherwise, Welch’s correction was applied. For comparisons among 3 or more independent groups at a single time point, one-way analysis of variance (ANOVA) followed by Tukey’s post hoc test was used. For longitudinal measurements, 2-way repeated-measures ANOVA followed by multiple-comparison correction was used. Survival curves were generated using the Kaplan–Meier method and compared by the log-rank test. A *P* value of <0.05 was considered statistically significant.

## Results and Discussion

### Synthesis and physicochemical characterization

**Fig. 2.**
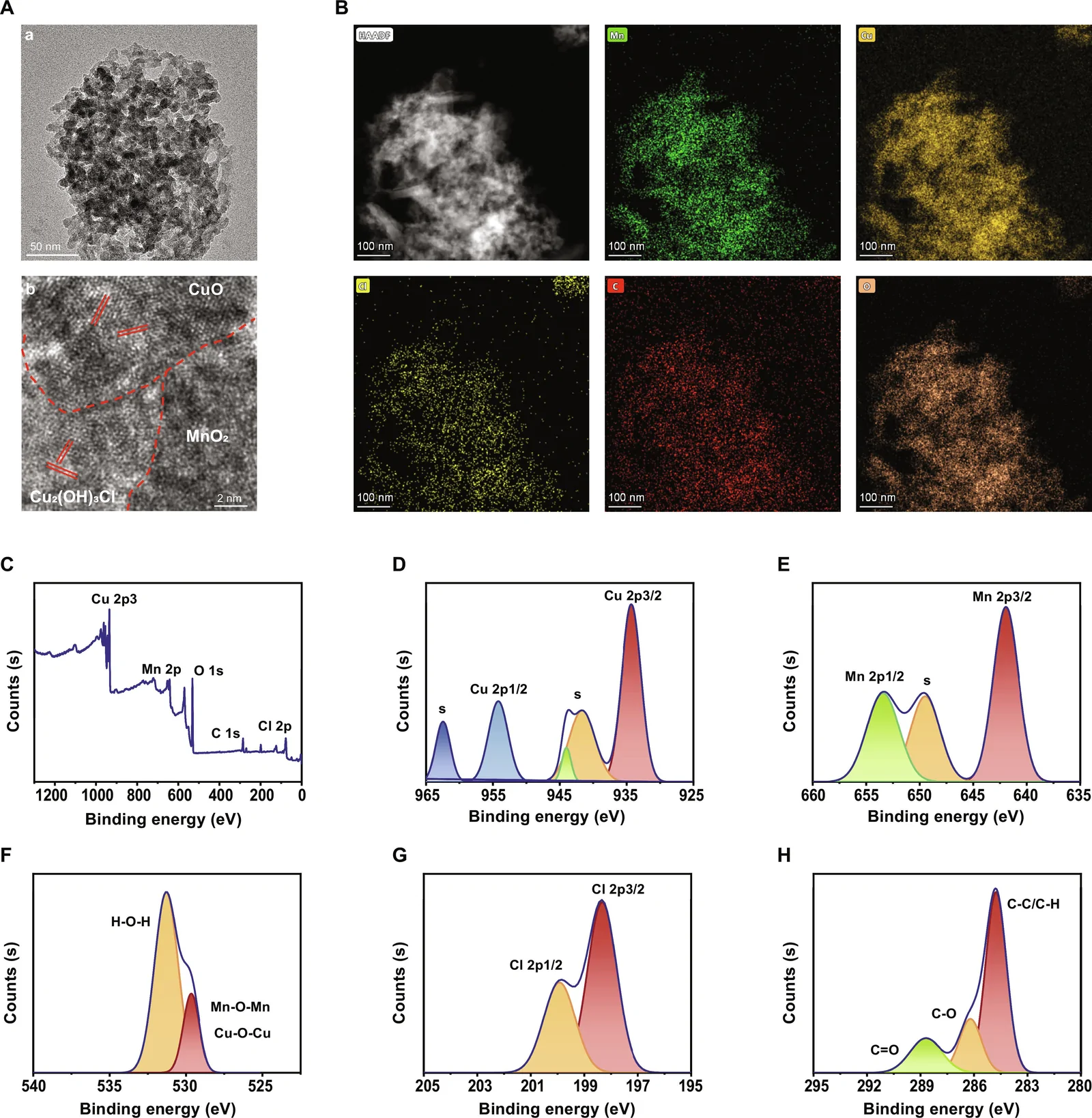
Synthesis and physicochemical characterization of CM. (A-a) TEM image of CM. Scale bar, 50 nm. (A-b) High-resolution TEM image of a single CM particle. Lattice spacings of 0.25 and 0.23 nm are assigned to CuO (1 1 −1) and (1 1 1) planes; lattice spacings of 0.27 and 0.25 nm are assigned to Cu_2_(OH)_3_Cl (2 0 1) and (0 4 0) planes; poorly resolved regions are attributed to amorphous MnO_2_. Scale bar, 2 nm. (B) EDS elemental mapping of CM (Mn, Cu, C, O, Cl). Scale bar, 100 nm. (C) XPS survey spectrum of CM. (D to H) High-resolution XPS spectra of Cu 2p (D), Mn 2p (E), O 1s (F), Cl 2p (G), and C 1s (H).

We engineered a CM designed to couple redox catalysis with copper-associated mitochondrial stress, thereby enabling tumor-selective cytotoxicity and microenvironment remodeling. Low-magnification TEM imaging showed that CM nanoparticles exhibited a relatively uniform projected size distribution (Fig. S1). Individual CM particles displayed a hierarchical morphology composed of smaller subparticle-like domains (Fig. 2A-a). This hierarchical “particle-of-subparticles” morphology is frequently associated with high interfacial area, which can increase exposure of reactive surface sites and facilitate catalytic turnover in aqueous media [17,18]. DLS analysis showed a hydrodynamic diameter of 141.8 nm with a PDI of 0.296 in normal aqueous dispersion, and the zeta potential was 29.3 ± 5.69 mV (Fig. S2A). In serum-containing solution, the hydrodynamic diameter increased to 190.0 nm, while the PDI remained 0.231 (Fig. S2B), suggesting moderate colloidal dispersity and serum-associated hydration/protein-corona formation rather than severe uncontrolled aggregation. HRTEM resolved distinct lattice fringes located in different regions within single particles (Fig. 2A-b), indicating multi-phase integration rather than a simple core–shell structure. In the upper region, lattice spacings of 0.25 and 0.23 nm were indexed to the (1 1 −1) and (1 1 1) planes of CuO (tenorite). In the lower left region, lattice spacings of 0.27 and 0.25 nm corresponded to the (2 0 1) and (0 4 0) planes of Cu_2_(OH)_3_Cl (atacamite). The lower right region displayed disordered and poorly resolved fringes consistent with amorphous MnO_2_. XRD further showed no intense sharp diffraction peaks, supporting the predominantly amorphous or low-crystallinity nature of the bulk CM structure (Fig. S3). These data support that CM comprises crystalline CuO, crystalline Cu_2_(OH)_3_Cl, and amorphous MnO_2_ coexisting within a single nanoparticle, a composition that is mechanistically relevant because Cu- and Mn-oxides can participate in complementary redox pathways, while phase boundaries can promote electron transfer and reactive intermediate formation [19–21].

EDS elemental mapping confirmed the presence of Mn, Cu, C, O, and Cl and demonstrated uniform distribution across nanoparticles (Fig. 2B). This spatial colocalization supported a blended microstructure rather than macroscopic segregation of Cu- and Mn-containing domains. Such blending is often desirable for catalytic nanozymes because it increases the probability of coupled redox cycling and can stabilize reactive intermediates by providing multiple surface adsorption environments [22,23]. XPS further confirmed the elemental composition and the expected valence states (Fig. 2C to H). Cu 2p3/2 and Cu 2p1/2 peaks at 934.28 and 954.18 eV, together with satellite peaks at 941.67, 944.09, and 962.45 eV, supported Cu(II) (Fig. 2D). Mn 2p3/2 and Mn 2p1/2 peaks at 641.95 and 653.43 eV with a satellite peak at 649.48 eV supported Mn(IV) (Fig. 2E). O 1s peaks at 529.67 and 531.30 eV were assigned to lattice oxygen and hydroxyl groups, respectively (Fig. 2F), indicating oxide surfaces with hydroxylated sites that can participate in H_2_O_2_ activation and thiol interactions [24]. Cl 2p3/2 and Cl 2p1/2 peaks at 198.33 and 199.93 eV supported Cl(−1) (Fig. 2G), consistent with the atacamite phase identified by HRTEM. C 1s peaks (284.76, 286.20, and 288.69 eV) were assigned to adventitious carbon and surface carbonaceous species, including C–C/C–H, C–O, and C=O components, and were not interpreted as intentional structural components of CM (Fig. 2H). The microscopy and surface spectroscopy define CM as a multi-component Cu(II)/Mn(IV) nanozyme with mixed crystallinity and a uniformly integrated elemental architecture, providing a physicochemical basis for multi-pathway catalytic activity in biologically relevant aqueous conditions.

The acid-responsive release behavior of CM was then examined by inductively coupled plasma (ICP) analysis. Both Mn and Cu showed limited release under neutral pH 7.4 conditions, whereas markedly enhanced release was observed at pH 5.0 (Fig. S4A and B). This pH-dependent profile suggests that CM remains relatively stable under neutral conditions but becomes more labile in acidic environments, which may facilitate intracellular metal availability after uptake into acidic endosomal/lysosomal compartments. Such acid-responsive metal release provides an additional mechanistic layer linking the material composition of CM to redox catalysis and copper-associated mitochondrial stress in tumor cells.

### Catalytic activity under tumor-relevant conditions

**Fig. 3.**
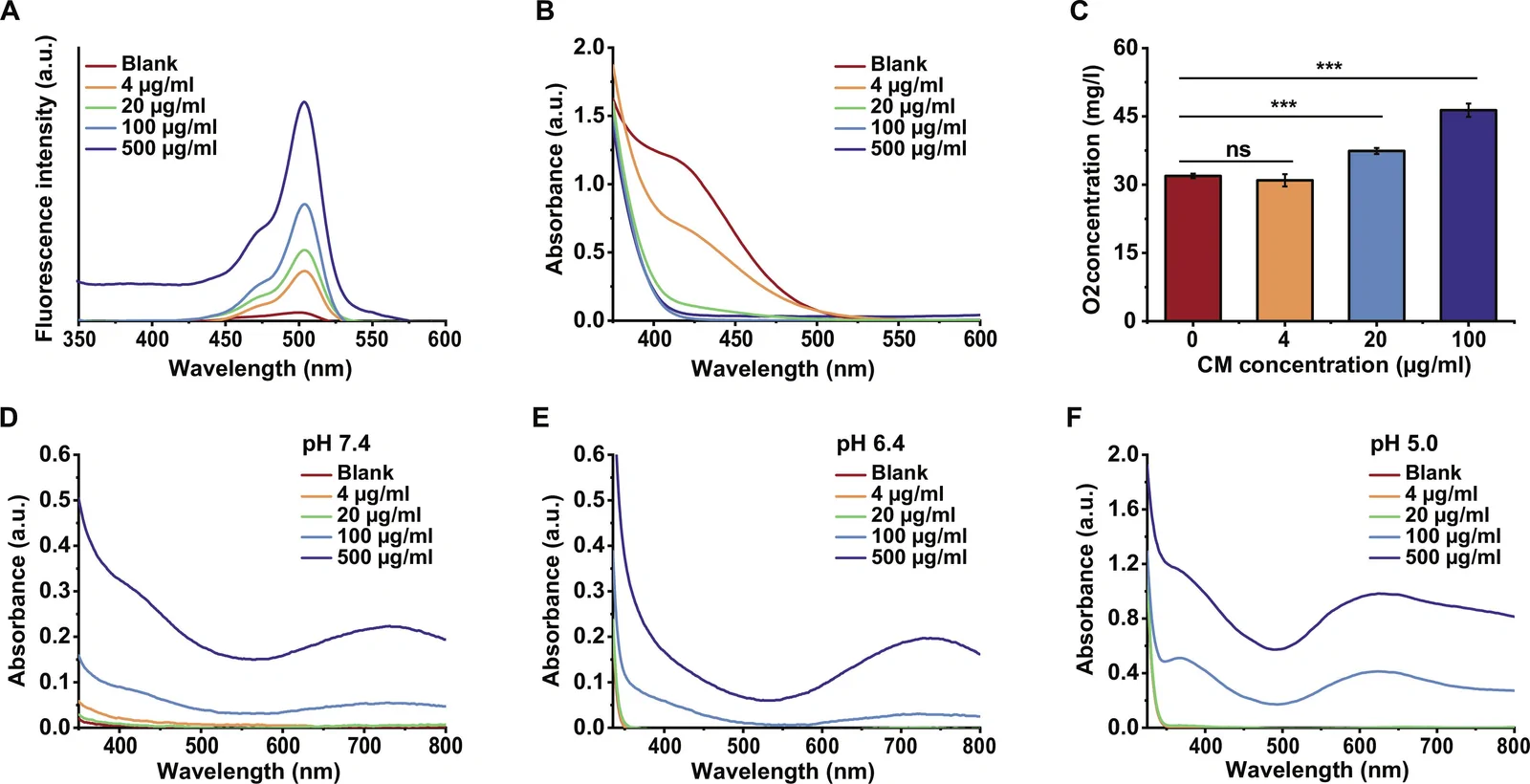
Multi-enzyme-mimetic catalytic activities of CM. (A) Peroxidase-like activity measured by DCFH fluorescence in the presence of H_2_O_2_ with CM at the indicated concentrations. (B) GSH oxidase-like activity measured by DTNB assay with CM at the indicated concentrations. (C) Catalase-like activity measured by dissolved oxygen generation from H_2_O_2_ with CM at the indicated concentrations. Data are presented as mean ± SD. Statistical significance was assessed by one-way ANOVA. *P < 0.05, **P < 0.01, ***P < 0.001, ****P < 0.0001. (D to F) TMB oxidation assay for Fenton-like activity of CM in the presence of H_2_O_2_ under graded pH conditions: pH 7.4 (D), pH 6.4 (E), and pH 5.0 (F).

To connect structure with function, we next evaluated catalytic activities that were directly relevant to tumor redox biology. CM exhibited peroxidase-like activity in the presence of H_2_O_2_, as demonstrated by the concentration-dependent increase of DCFH fluorescence (Fig. 3A). This result indicates that CM can promote ROS generation when H_2_O_2_ is available, a condition often present in tumors due to metabolic reprogramming and inflammatory signaling [25,26]. In parallel, CM displayed GSH oxidase-like activity in DTNB assay, where absorbance decreased with increasing CM concentration (Fig. 3B), indicating progressive GSH consumption. GSH depletion is mechanistically important because it reduces the capacity to buffer ROS and disrupts protein thiol homeostasis, thereby sensitizing cells to oxidative injury and mitochondrial dysfunction [27–29].

CM also showed catalase-like activity, producing O_2_ from H_2_O_2_ in a concentration-dependent manner as measured by a dissolved oxygen meter (Fig. 3C). In addition, CM displayed acidity-activated Fenton-like behavior, as evidenced by TMB oxidation under graded pH conditions (Fig. 3D to F). The oxidation signal was strongest at pH 5.0, whereas signals at pH 6.4 and pH 7.4 were comparatively low and similar, indicating that acidic conditions favor TMB-oxidizing reactive conversion by CM. Importantly, pH 5.0 was used to model acidic endosomal/lysosomal compartments after cellular uptake rather than the bulk peritoneal fluid, while pH 6.4 was included to approximate the mildly acidic extracellular tumor milieu [30,31]. Across all pH conditions, increasing CM concentration further enhanced the oxidation signal, consistent with a concentration-dependent catalytic response. The acidic preference is aligned with microenvironment-tuned catalysis, as modest acidity is a common feature in solid tumors and peritoneal implants [32,33].

To provide quantitative kinetic parameters, we further performed apparent kinetic analysis based on the Lineweaver–Burk double-reciprocal plots for catalase- and peroxidase-like activities using H_2_O_2_ as the substrate (Fig. S5A and B). For catalase-like activity, CM showed an apparent *K*_m_ of 337.6 mM and an apparent *V*_max_ of 0.624 mM s^−1^. For peroxidase-like activity, CM showed an apparent *K*_m_ of 38 mM and an apparent *V*_max_ of 4.02 × 10^−6^ M s^−1^. These kinetic parameters demonstrate measurable H_2_O_2_-dependent catalytic turnover and provide a quantitative basis for comparing CM with previously reported nanozymes. Compared with representative nanozymes in Table S1, CM exhibited a relatively high apparent *V*_max_ and favorable *V*_max_/*K*_m_ for catalase-like H_2_O_2_ decomposition, indicating efficient H_2_O_2_ processing and O_2_ generation. CM also showed a high apparent *V*_max_ for peroxidase-like H_2_O_2_-mediated TMB oxidation, suggesting rapid oxidative conversion. These comparisons are considered apparent benchmarks due to variations in experimental conditions.

The combined catalytic profile supports a compartment-dependent H_2_O_2_-processing model. Catalase (CAT)- and peroxidase (POD)/Fenton-like reactions both involve H_2_O_2_, with their contributions governed by local pH, GSH availability, substrate access, and intracellular localization [34,35]. Near-neutral or mildly acidic extracellular conditions favor catalase-like H_2_O_2_ decomposition and O_2_ generation, while more acidic endosomal/lysosomal environments promote Fenton-like oxidative conversion. Meanwhile, GSH consumption weakens antioxidant buffering and increases tumor cell sensitivity to oxidative and mitochondrial stress. CM thus functions as a pH- and substrate-dependent redox-modulating nanozyme.

### Cellular uptake and 3-dimensional penetration

**Fig. 4.**
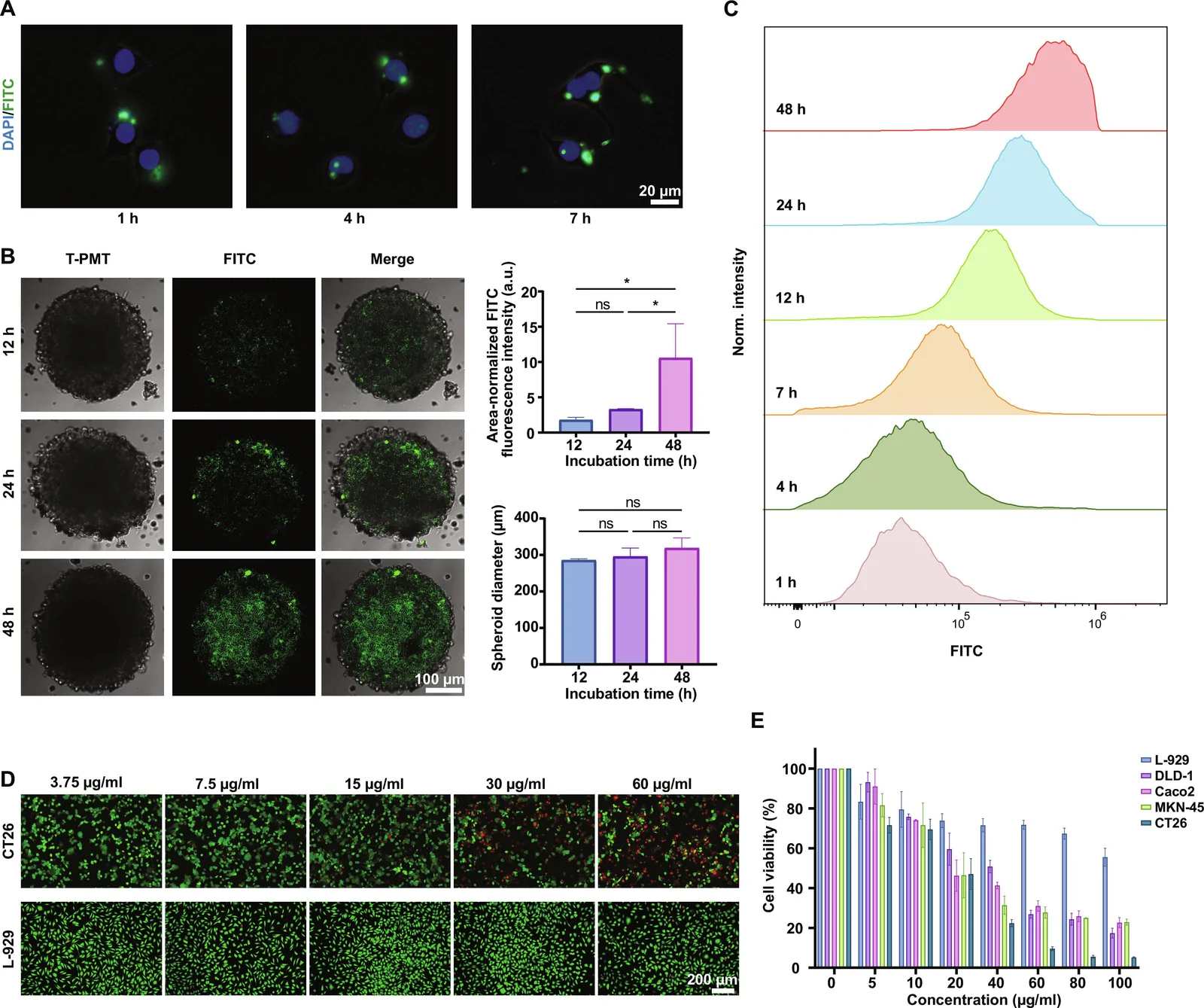
Cellular uptake, spheroid penetration, and tumor-selective cytotoxicity. (A) Representative fluorescence microscopy images of CT26 cells incubated with FITC-CM for the indicated times. Scale bar, 20 μm. (B) CLSM images of size-controlled CT26 multicellular tumor spheroids after incubation with FITC-CM for 12, 24, and 48 h. Left panel: Representative fluorescence distribution within spheroids. Upper right panel: Quantification of area-normalized FITC fluorescence intensity. Lower right panel: Spheroid diameter measurements confirming comparable sizes among groups. Scale bar, 100 μm. (C) Flow cytometry quantification of FITC-CM uptake in CT26 cells over 1 to 48 h. (D) Live/dead staining (Calcein-AM/PI) of CT26 and L929 cells treated with CM at the indicated concentrations. Scale bar, 200 μm. (E) CCK-8 cell viability of CM (0 to 100 μg/ml) in MKN-45, DLD-1, Caco2, CT26, and L929 cells. Data are presented as mean ± SD. Statistical significance was assessed by one-way ANOVA. *P < 0.05, **P < 0.01, ***P < 0.001, ****P < 0.0001.

IP therapy for peritoneal metastasis requires that materials not only remain within the peritoneal cavity but also enter tumor cells and distribute within compact tumor nodules [36,37]. To examine cellular internalization, CM was labeled with FITC and incubated with CT26 cells (FITC-CM). Fluorescence microscopy showed time-dependent enhancement of green fluorescence within tumor cells, with prominent intracellular signal after 7 h (Fig. 4A), indicating efficient uptake. Flow cytometry from 1 to 48 h confirmed progressive accumulation of FITC fluorescence with extended incubation time, showing a linear correlation between exposure duration and cellular uptake (Fig. 4C). This time-dependent uptake provides a mechanistic bridge between the catalytic surface chemistry of CM and the intracellular mitochondrial and redox phenotypes observed later, as mitochondrial stress programs generally require sufficient intracellular availability of catalytic or metal-active components [11,15].

We then examined whether CM could penetrate 3-dimensional (3D) tumor architecture using CT26 multicellular tumor spheroids, which model diffusion-limiting conditions relevant to peritoneal implants [38]. Bright-field measurements confirmed that spheroid diameters were comparable among the 12, 24, and 48 h groups, excluding spheroid size as a major confounder for fluorescence penetration analysis (Fig. 4B, lower right panel). CLSM showed a stepwise penetration profile. FITC fluorescence was detectable at 12 h, increased at 24 h with enhanced peripheral and inner-layer signals, and became strong and uniformly distributed throughout the spheroid by 48 h (Fig. 4B, left panel). Quantitative analysis of area-normalized FITC fluorescence (Fig. 4B, upper right panel) further demonstrated a time-dependent increase in intratumoral signal, reaching a maximum at 48 h. This progressive inward distribution indicates that CM is not confined to the outer rim of the spheroid and supports its ability to access cells within deeper regions of tumor-like tissue. Such penetration behavior is particularly relevant for peritoneal nodules, where dense extracellular matrix and elevated interstitial pressure commonly restrict chemotherapy drug transport and limit therapeutic depth [39,40].

### Tumor-selective cytotoxicity across cell lines

We next evaluated whether the catalytic and delivery properties translated into cytotoxic activity and whether cytotoxicity displayed tumor selectivity. A CCK-8 assay was conducted using increasing concentrations of CM (0 to 100 μg/ml) in multiple cancer cell lines, including human gastric cancer (MKN-45), CRC (DLD-1), rectal cancer (Caco2), and murine colon carcinoma (CT26), alongside normal mouse fibroblasts (L929). All tumor cell lines exhibited dose-dependent viability reduction, with CT26 showing the greatest sensitivity (IC_50_ = 15.49 μg/ml). In contrast, L929 cells retained high viability even at 100 μg/ml (IC_50_ = 366.8 μg/ml) (Fig. 4E and Fig. S6). This large differential is compatible with a redox-amplification mechanism, as many tumor cells operate under higher basal oxidative pressure and rely more heavily on antioxidant systems for survival, which can increase vulnerability to ROS elevation and thiol depletion [26,29].

Live/dead staining (Calcein-AM/PI) further supported selective cytotoxicity. CT26 cells showed extensive PI-positive staining at concentrations ≥15 μg/ml, while L929 cells remained predominantly viable across 3.75 to 60 μg/ml (Fig. 4D). Based on both sensitivity and direct relevance to the in vivo peritoneal metastasis model, CT26 was selected for downstream mechanistic and therapeutic experiments.

### Mechanistic insights into ROS amplification and cuproptosis

**Fig. 5.**
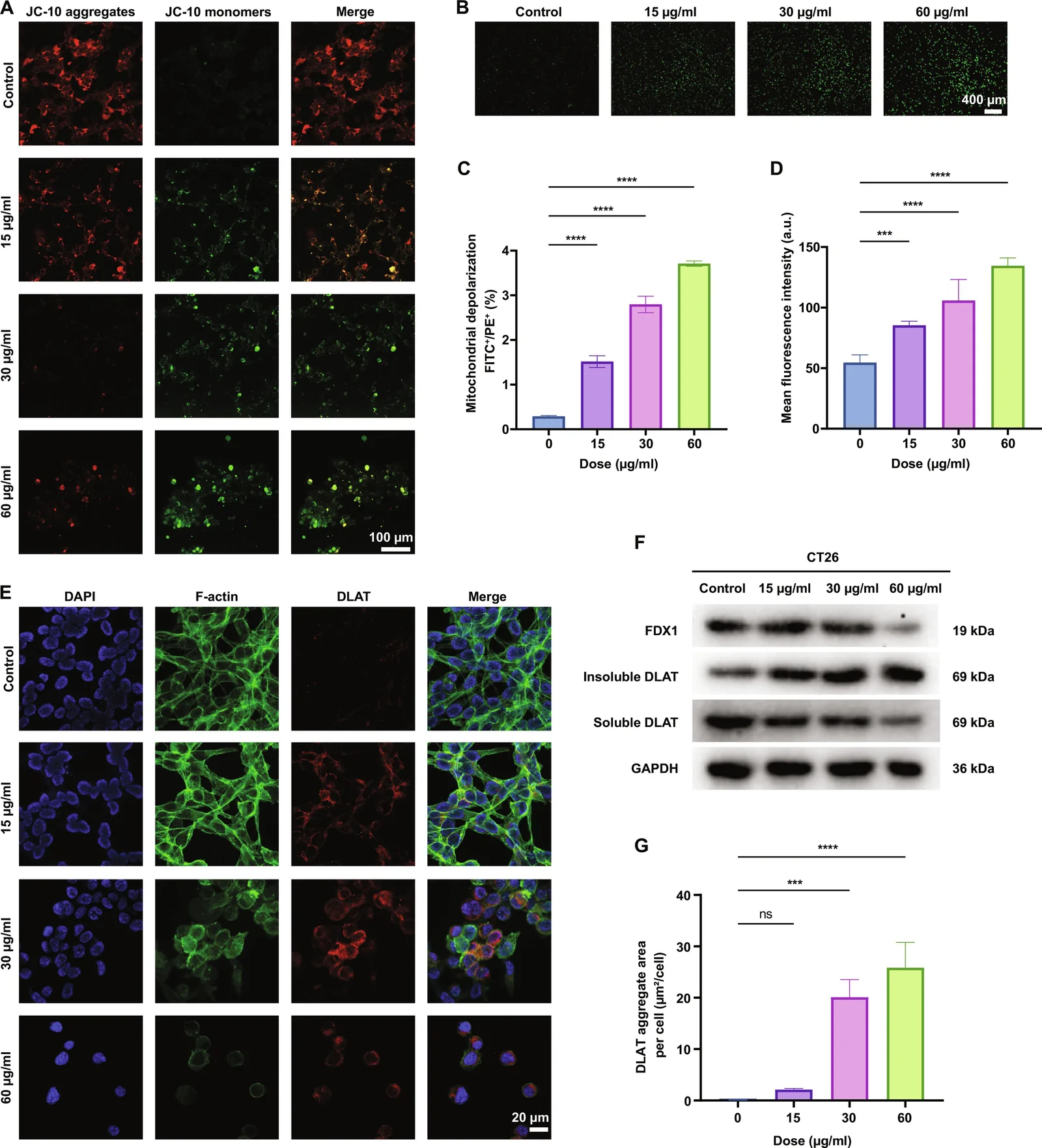
ROS amplification, mitochondrial depolarization, and cuproptosis-associated signatures. (A) JC-10 fluorescence images of CT26 cells treated with CM (15, 30, and 60 μg/ml). Scale bar, 100 μm. (B) DCFH-DA fluorescence images of CT26 cells treated with CM at the indicated concentrations. Scale bar, 400 μm. (C) Flow cytometry quantification of mitochondrial membrane potential based on the JC-10 red/green ratio under the indicated treatments. (D) Quantification of intracellular ROS based on DCFH-DA fluorescence microscopy. (E) Immunofluorescence staining of DLAT in CT26 cells after CM treatment at the indicated concentrations. Scale bar, 20 μm. (F) Immunoblot analysis of FDX1 and detergent-soluble/insoluble DLAT fractions in CT26 cells following CM treatment at the indicated concentrations. (G) Quantification of DLAT aggregate area per cell based on immunofluorescence image analysis. Data are presented as mean ± SD. Statistical significance was assessed by one-way ANOVA. *P < 0.05, **P < 0.01, ***P < 0.001, ****P < 0.0001.

Given the observed catalytic activity and tumor-selective cytotoxicity, we investigated cellular phenotypes that link nanozyme redox chemistry to regulated cell death programs. MMP was assessed using JC-10 staining after CM treatment (15, 30, and 60 μg/ml). A dose-dependent mitochondrial depolarization phenotype was observed, with pronounced red-to-green fluorescence shift at 30 and 60 μg/ml (Fig. 5A). Flow cytometry confirmed a substantial reduction in the red/green ratio (Fig. 5C), indicating disruption of MMP and mitochondrial functional collapse.

Intracellular ROS was quantified using DCFH-DA staining. Microscopy showed increased green fluorescence in CM-treated cells at intermediate and high concentrations (Fig. 5B). Quantification of mean fluorescence intensity confirmed elevated ROS compared to the untreated control (Fig. 5D). In addition, intracellular GSH analysis showed that CM reduced cellular GSH levels in a time- and concentration-dependent manner, with marked depletion after prolonged exposure (Fig. S7). These cellular data connect to the solution-phase assays by demonstrating that CM can amplify oxidative stress in a biological context, supporting a mechanism in which CM catalyzes ROS formation while simultaneously weakening antioxidant buffering, thereby pushing tumor cells beyond their tolerable redox threshold. In the setting of IP metastasis, such redox destabilization can be particularly relevant because ascites and inflammatory infiltrates can provide H_2_O_2_ and redox-active mediators that feed catalytic cycles [41,42].

To evaluate whether CM engages copper-dependent mitochondrial proteotoxic stress consistent with the cuproptosis framework, we examined DLAT behavior and FDX1 expression. Immunofluorescence staining revealed punctate DLAT aggregates in the cytoplasm of cells exposed to CM, with aggregation intensity increasing in a dose-dependent manner (Fig. 5E). In the DLAT immunofluorescence assay, F-actin staining was used to visualize cytoskeletal morphology and cell boundaries. The weakened and disorganized F-actin signal observed after high-dose CM treatment reflects cytoskeletal disruption and reduced cell integrity under cytotoxic conditions, rather than serving as a normalization reference [43,44]. Quantification of DLAT-positive aggregate area normalized to cell number confirmed a concentration-dependent increase in DLAT aggregation (Fig. 5G). Western blotting showed reduced FDX1 expression and altered DLAT distribution after CM treatment (Fig. 5F and Fig. S8). Detergent-soluble/insoluble fractionation demonstrated increased DLAT abundance in the insoluble fraction following CM exposure, supporting DLAT aggregation rather than a simple redistribution between fractions (Fig. 5F). DLAT aggregation/oligomerization and FDX1 involvement have been described as core molecular features of copper-triggered cell death centered on lipoylated tricarboxylic acid (TCA) cycle components [11,45,46]. Within the evidence presented here, CM induces a combined phenotype of mitochondrial depolarization, ROS elevation, and cuproptosis-associated protein signatures, providing mechanistic consistency between CM’s copper-containing catalytic design and the observed tumor cell killing.

### In vivo safety, IP retention, and biodistribution

**Fig. 6.**
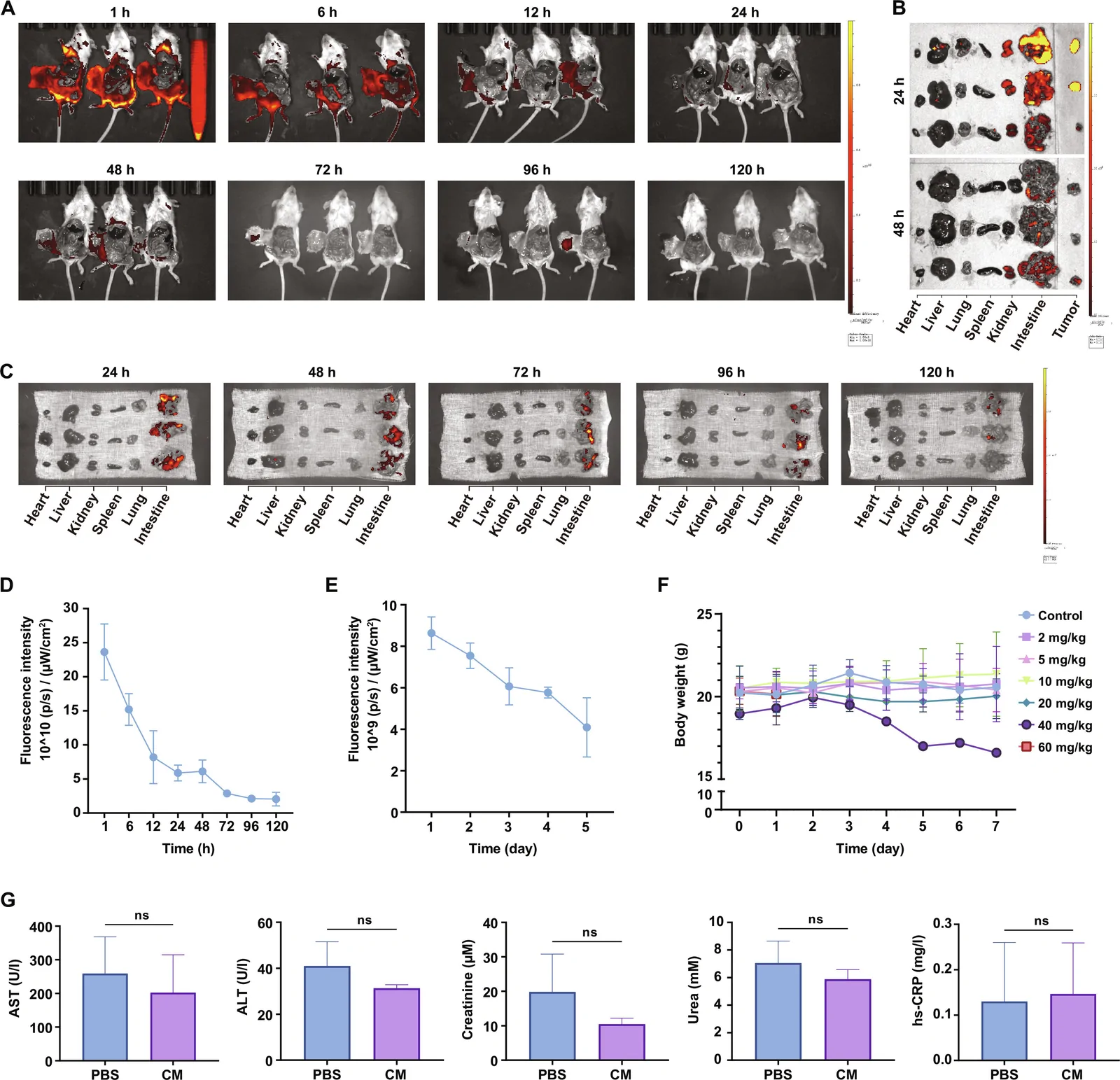
IP retention, biodistribution, and biosafety of CM in vivo. (A) In vivo IVIS fluorescence imaging of the abdomen following IP administration of FITC-CM at the indicated time points. (B) Ex vivo IVIS fluorescence imaging of dissected major abdominal organs and peritoneal metastatic nodules at the indicated time points after IP FITC-CM administration. (C) Ex vivo fluorescence imaging of major abdominal organs at the indicated time points after IP FITC-CM administration. (D) Quantification of abdominal fluorescence signal over time. (E) Quantification of ex vivo intestinal wall-associated fluorescence intensity at the indicated time points. (F) Body weight changes over 7 d in mice following IP administration of CM at the indicated doses (0 to 60 mg/kg). (G) Serum biochemistry indices for mice treated with CM (20 mg/kg) and control mice. Data are presented as mean ± SD. Statistical significance was assessed by unpaired 2-tailed Student’s *t* test. *P < 0.05, **P < 0.01, ***P < 0.001, ****P < 0.0001.

For clinical translation of IP administration, tolerability and peritoneal residence kinetics are central to defining a feasible dosing schedule. BALB/c mice received IP injections of increasing CM doses (0 to 60 mg/kg). Weight loss and mortality were observed at doses above 20 mg/kg, whereas mice receiving ≤20 mg/kg maintained stable body weights and survived without adverse effects (Fig. 6F and Fig. S9). Based on this in vivo tolerability profile, 20 mg/kg was selected as the working dose for subsequent studies.

To examine IP retention and biodistribution, FITC-CM was administered IP and monitored by IVIS. Abdominal fluorescence decreased over time, remained detectable at 48 h, and was nearly eliminated by 72 h (Fig. 6A and D). Ex vivo imaging of major abdominal organs revealed predominant retention in the intestinal wall up to 96 h post-injection (Fig. 6C and E), indicating prolonged peritoneal residency with organ-adjacent exposure. In a CT26 peritoneal metastasis model, IVIS revealed strong tumor-localized fluorescence at 24 h and markedly reduced signals by 48 h (Fig. 6B), while intestinal retention persisted. These kinetics suggest that CM can access tumor sites after IP delivery and support the operational choice of a 48-h dosing interval for therapy studies. Serum biochemistry analysis showed no significant differences between treated and control groups in liver and kidney function or inflammatory markers (Fig. 6G), supporting a favorable systemic safety profile at the selected regimen. Consistently, H&E staining of major organs (heart, liver, spleen, lung, and kidney) from mice treated at 20 mg/kg CM showed no overt histopathological damage, inflammatory infiltration, or tissue necrosis (Fig. S10). In addition, peritoneal tissue sections did not show obvious inflammatory damage or persistent particulate residues at the examined endpoint, supporting local peritoneal tolerability. Collectively, these findings establish a practical IP dosing framework that aligns exposure kinetics with treatment scheduling and downstream efficacy testing.

### Antitumor efficacy in peritoneal metastasis and immune remodeling

**Fig. 7.**
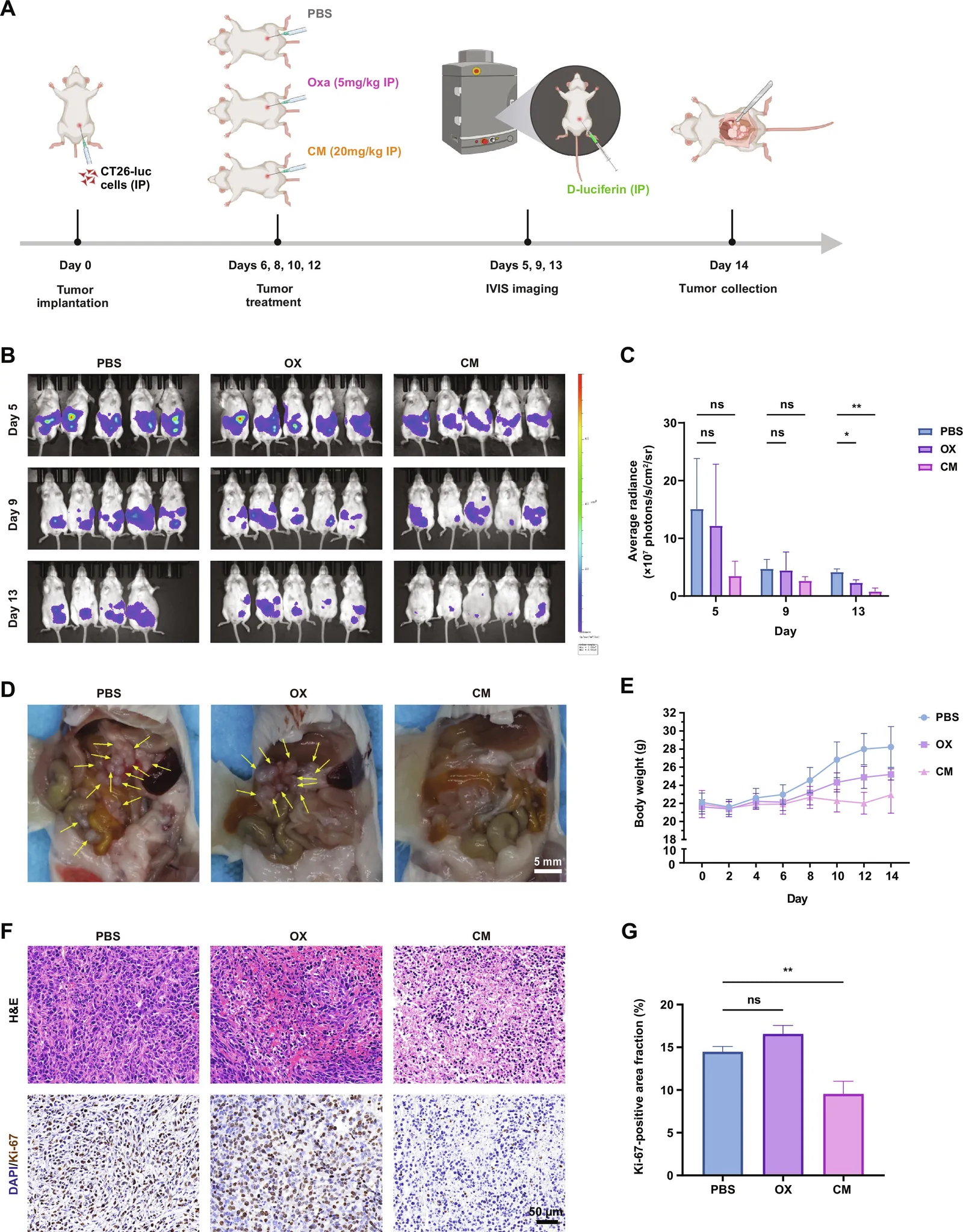
Antitumor efficacy of CM in CT26-luc peritoneal metastasis model. (A) Schematic of the CT26-luc peritoneal metastasis model and treatment timeline. CT26-luc cells were injected IP on day 0. Tumor establishment was evaluated by IVIS on day 5, followed by randomization and IP treatments (PBS, OX, or CM) every 48 h on days 6, 8, 10, and 12. IVIS was performed on days 9 and 13, and mice were euthanized for tissue collection on day 14. (B) Longitudinal IVIS bioluminescence images of mice from each treatment group at the indicated time points. (C) Quantification of bioluminescence signal intensity (*n* = 5 per group, except PBS *n* = 4 at endpoint). Statistical significance was assessed by 2-way repeated-measures ANOVA. *P < 0.05, **P < 0.01, ***P < 0.001, ****P < 0.0001. (D) Representative gross images of the peritoneal cavity at endpoint. Yellow arrows indicate peritoneal metastatic nodules. Scale bar, 5 mm. (E) Body weight changes during treatment. (F) Representative histological images of peritoneal tumor tissues across groups: H&E staining (top) and Ki-67 immunohistochemistry (bottom). Scale bar, 50 μm. (G) Quantification of Ki-67 immunohistochemistry as Ki-67-positive area fraction. Data are presented as mean ± SD. Statistical significance was assessed by one-way ANOVA. *P < 0.05, **P < 0.01, ***P < 0.001, ****P < 0.0001.

We evaluated therapeutic efficacy in a CT26-luciferase peritoneal metastasis model (Fig. 7A). IVIS imaging on day 5 confirmed successful tumor establishment with comparable baseline signals among groups. On days 9 and 13, CM-treated mice showed significantly reduced tumor fluorescence intensity compared with both PBS and OX groups (Fig. 7B and C). A decrease in fluorescence was also observed in the PBS group at day 13, likely due to extensive ascites and tumor burden interfering with luciferin uptake and tumor access. Such imaging behavior has been reported in ascites-dominant IP tumor settings and supports interpreting late-stage bioluminescence imaging signals together with orthogonal endpoints [47].

Gross anatomical examination substantiated the therapeutic effect, revealing widespread tumor nodules and ascites in control mice, whereas the CM group showed minimal or no visible peritoneal lesions (Fig. 7D and Fig. S11). Body weight trends further supported reduced ascites-associated burden. PBS mice exhibited weight gain attributable to ascites accumulation, while CM-treated mice maintained stable weights (Fig. 7E). Histology of tumor tissues showed extensive necrosis and reduced cellularity in the CM group, and Ki-67 immunohistochemistry showed a marked reduction in proliferative index compared to PBS and OX groups (Fig. 7F and G). These results link longitudinal imaging to tissue-level evidence of tumor suppression and provide a consistent picture of antitumor activity under an IP dosing schedule selected based on retention kinetics.

**Fig. 8.**
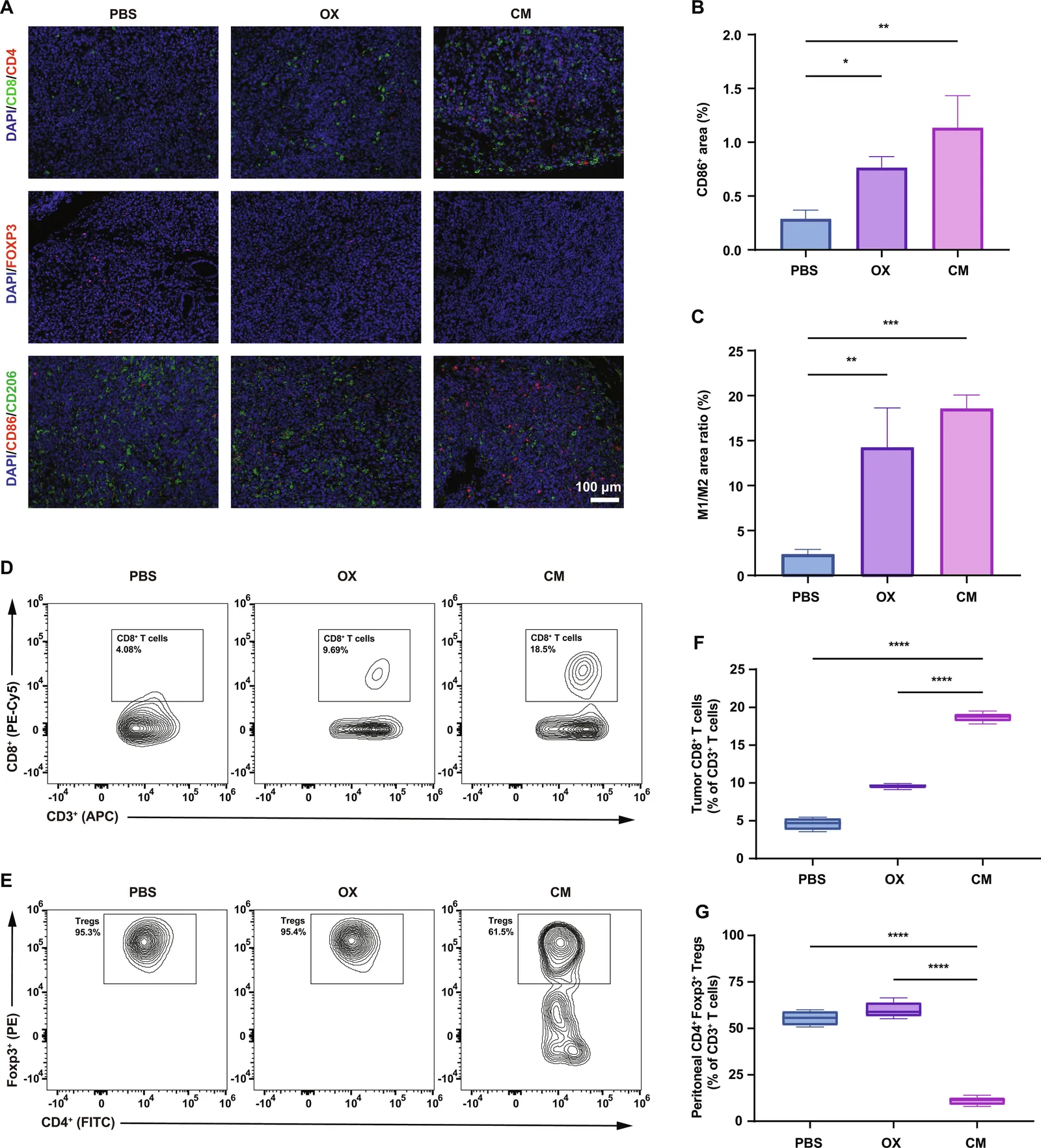
Immune remodeling in peritoneal tumors after CM therapy. (A) Representative immunofluorescence images of peritoneal tumor sections across treatment groups: CD4 and CD8 staining (top), Foxp3 staining (middle), and CD86 and CD206 staining (bottom). Scale bar, 100 μm. (B) Quantification of CD86^+^ immunofluorescence-positive area in tumor sections. (C) M1/M2 ratio derived from CD86^+^ and CD206^+^ immunofluorescence-positive area quantification. (D) Flow cytometry plots for tumor CD3^+^CD8^+^ T cells. (E) Flow cytometry plots for CD4^+^Foxp3^+^ Tregs in the peritoneal compartment. (F) Quantitative analysis of tumor CD8^+^ T cells. (G) Quantitative analysis of peritoneal Tregs. Data are presented as mean ± SD. Statistical significance was assessed by one-way ANOVA. *P < 0.05, **P < 0.01, ***P < 0.001, ****P < 0.0001.

Given the increasing appreciation that peritoneal metastasis is sustained by an immunosuppressive niche [48], we next assessed whether CM affected immune composition within peritoneal tumors. Immunofluorescence and flow cytometry demonstrated increased CD8^+^ cytotoxic T cell infiltration in the CM group and a modest elevation of CD4^+^ helper T cells. Conversely, Foxp3^+^ Tregs were abundant in PBS and OX groups but markedly reduced in the CM group (Fig. 8A). The flow cytometry gating strategies are provided in Fig. S12. Flow cytometry confirmed these trends quantitatively. In tumors, CM treatment significantly increased CD8^+^ T cell frequencies (*P* < 0.0001; Fig. 8D and F). In the peritoneal compartment, CM-treated mice exhibited significantly reduced Foxp3^+^ Treg frequencies (*P* < 0.001; Fig. 8E and G). In contrast, peritoneal CD8^+^ T cell frequencies did not differ significantly among the 3 groups (Fig. S13A). Tumor-infiltrating Tregs were reduced in both the OX and CM groups relative to controls, with no clear difference between OX and CM (Fig. S13B). These immune shifts align with an antitumor immune balance favoring cytotoxic effector presence and reduced regulatory suppression in the peritoneal tumor microenvironment.

In peritoneal metastasis, M2-like macrophages can contribute to immune suppression and tumor support through cytokine programs and checkpoint ligand expression, whereas M1 skewing is more compatible with inflammatory antigen processing and effector recruitment [48]. In the CM group, macrophage polarization was skewed toward an M1-like phenotype. Tumor sections from the CM group exhibited increased CD86^+^ (M1) and decreased CD206^+^ (M2) macrophage infiltration, with quantification indicating an increased M1/M2 ratio (Fig. 8A to C). Mechanistically, oxidative and mitochondrial stress can promote inflammatory signaling and reshape myeloid polarization programs [49]. In our dataset, CM reduced tumor burden and concomitantly remodeled the immune landscape, with increased CD8^+^ T cell enrichment, reduced Treg abundance, and a shift toward an M1-like macrophage phenotype. These changes coincide with CM-driven catalytic stress and copper-associated mitochondrial disruption, extending the effect of CM beyond direct tumor cell killing to reshaping the peritoneal immune niche.

## Conclusion

In this study, we developed a copper–manganese nanozyme composed of crystalline CuO and Cu_2_(OH)_3_Cl integrated with amorphous MnO_2_ in a uniformly colocalized architecture. CM was designed to integrate tumor-relevant Fenton-like redox catalysis with copper-driven mitochondrial proteotoxic stress, thereby combining oxidative damage with a cuproptosis-associated death program. In tumor cells, CM promoted ROS accumulation together with depletion of thiol-based antioxidant buffering and disruption of mitochondrial homeostasis, while normal fibroblasts showed substantially higher tolerance. This integrated mechanism translated into robust therapeutic efficacy in an IP metastasis model, with marked suppression of peritoneal tumor growth and reduced ascites-associated burden compared with controls. CM treatment also remodeled the peritoneal tumor microenvironment, featuring increased CD8^+^ T cell infiltration, decreased Foxp3^+^ Treg enrichment, and a macrophage shift toward an M1-dominant phenotype. CM displayed good IP tolerability at the working regimen and provided a practical exposure window compatible with repeated dosing. Together, these findings define CM as an IP nanozyme platform that couples Fenton-like catalytic therapy with cuproptosis-linked mitochondrial disruption and immune reprogramming, supporting its further development as a potential treatment strategy for peritoneal metastasis.

## Data Availability

The data supporting the findings of this study are available from the corresponding authors upon reasonable request.

## Supplementary Materials

Supplementary 1 Figs. S1 to S13 Table S1 References [50–59]
