# Supplementary material for: Intraperitoneal Copper–Manganese Nanozyme Suppresses Peritoneal Metastasis with Antitumor Immune Activation
Source: Biomater Res. 2026 Sep 9;30:0401. doi: 10.34133/bmr.0401 (PMC13554416; doi:10.34133/bmr.0401)
Supplement: Supplementary file 1 [file bmr.0401.f1.docx]

Supplementary Materials

Intraperitoneal copper-manganese nanozyme suppresses peritoneal metastasis with antitumor immune activation

*Jinquan Zhang^1,†^, Zhenze Xie^2,3,†^, Muye He^2,3^, Siqi He^4^, Ling Wang^2,3^, Chengxiao Song^2,3^, Jingguang Wang^2,3^, Jian Li^1,*^, Chang Du^2,3,*^, Ruoxu Dou^4,*^*

^1^ Department of Hepatobiliary Surgery, The Fifth Affiliated Hospital, Sun Yat-Sen University, Zhuhai, Guangdong, 519000, PR China

^2^ Department of Biomedical Engineering, School of Materials Science and Engineering, South China University of Technology, Guangzhou, Guangdong, 510641, PR China

^3^ National Engineering Research Center for Tissue Restoration and Reconstruction, South China University of Technology, Guangzhou, Guangdong, 510006, PR China

^4^ Comprehensive Cancer Center, Foresea Life Insurance Guangzhou General Hospital, Guangzhou, Guangdong, 511300, P. R. China

^†^ Jinquan Zhang and Zhenze Xie contributed equally to this work.

^*^ Address correspondence to: Jian Li (lijian5@mail.sysu.edu.cn); Chang Du (duchang@scut.edu.cn); Ruoxu Dou (dourx@mail.sysu.edu.cn).

Fig. S1. Low-magnification TEM characterization of CM. Representative low-magnification TEM image showing multiple CM nanoparticles and the overall morphology of the prepared nanozyme. CM displayed a relatively uniform projected particle size, although partial interparticle contact may occur during TEM sample preparation.

Fig. S2. Colloidal size distribution and surface charge of CM. (A) DLS size distribution and zeta potential of CM in normal aqueous dispersion. Hydrodynamic size = 141.8 nm; PDI = 0.296; zeta potential = 29.3 ± 5.69 mV. (B) DLS size distribution of CM in serum-containing solution. Hydrodynamic size = 190.0 nm; PDI = 0.231.

Fig. S3. XRD characterization of CM. XRD pattern of CM showing the absence of intense sharp diffraction peaks, supporting the predominantly amorphous or low-crystallinity nature of the bulk CM structure.

Fig. S4. pH-dependent Mn and Cu ion release from CM. (A) Mn ion release profiles of CM at pH 7.4 and pH 5.0. (B) Cu ion release profiles of CM at pH 7.4 and pH 5.0. Both Mn and Cu showed limited release under neutral conditions, whereas acidic conditions promoted metal-ion release. Under the tested release conditions, the cumulative released Cu/Mn molar ratio was approximately 2.16:1. This value reflects the released soluble metal fraction and should not be interpreted as the bulk stoichiometric Cu/Mn ratio of the solid CM nanozyme.

Fig. S5. Apparent enzyme kinetic analysis of CM. (A) Lineweaver-Burk double-reciprocal plot for catalase-like activity using H₂O₂ as the substrate. The apparent Km and Vmax values were calculated as 337.6 mM and 0.624 mM s⁻¹, respectively. (B) Lineweaver-Burk double-reciprocal plot for peroxidase-like activity using H₂O₂ as the substrate. The apparent Km and Vmax values were calculated as 38 mM and 4.02×10⁻⁶ M s⁻¹, respectively.

Table S1. Comparison of apparent catalytic kinetic parameters between CM and representative reported nanozymes.

| Material | Enzyme-like activity | Substrate | Km (mM) | Vmax (mM s^-1^) | | Vmax/Km (s^-1^) |
| --- | --- | --- | --- | --- | --- | --- |
| CM nanozyme | CAT-like | H_2_O_2_ | 3.38E+02 | | 0.624 | 1.85E-03 |
| MoCC SAzyme[50] | CAT-like | H_2_O_2_ | 3.72E+00 | | 7.88E-04 | 2.12E-04 |
| Fe-SANzyme[51] | CAT-like | H_2_O_2_ | 1.88E+01 | | 9.32E-03 | 4.96E-04 |
| Pt-Ft[52] | CAT-like | H_2_O_2_ | 4.21E+02 | | 0.84 | 2.00E-03 |
| Au-Si-ACD[53] | CAT-like | H_2_O_2_ | 1.17E+02 | | 1.18E-04 | 1.01E-06 |
| CN[54] | CAT-like | H_2_O_2_ | 3.93E+02 | | 2.48E-03 | 6.31E-06 |
| Co3O4 nanoplates[55] | CAT-like | H_2_O_2_ | 2.47E+01 | | 2.38E-03 | 9.64E-05 |
| Co3O4 nanorods[55] | CAT-like | H_2_O_2_ | 4.82E+00 | | 1.89E-03 | 3.92E-04 |
| Co3O4 nanocubes[55] | CAT-like | H_2_O_2_ | 6.39E+01 | | 1.23E-03 | 1.93E-05 |
| CM nanozyme | POD-like | H_2_O_2_ | 3.80E+01 | | 4.02E-03 | 1.06E-04 |
| MoCC SAzyme + US[50] | POD-like | H_2_O_2_ | 6.50E-01 | | 4.56E-04 | 7.02E-04 |
| Cu-SA-CN[56] | POD-like | H_2_O_2_ | 1.34E+00 | | 5.14E-04 | 3.84E-04 |
| TATP/TCPP-Fe[57] | POD-like | H_2_O_2_ | 2.10E-01 | | 8.30E-05 | 3.95E-04 |
| Mn/Fe SACs[58] | POD-like | H_2_O_2_ | 1.38E-01 | | 1.62E-04 | 1.17E-03 |
| HRP[58] | POD-like | H_2_O_2_ | 1.16E+01 | | 2.76E-05 | 2.37E-06 |
| Fe3O4 nanoparticles[59] | POD-like | H_2_O_2_ | 1.54E+02 | | 9.78E-05 | 6.35E-07 |

The apparent catalytic index was calculated as Vmax/Km after unit normalization. Because the reported kinetic parameters were obtained under different assay conditions, including nanozyme concentration, pH, buffer composition, substrate/probe system, temperature, and detection method, the comparison should be interpreted as an apparent kinetic benchmark rather than an absolute ranking of intrinsic catalytic activity.

Fig. S6. Dose-response curves and IC₅₀ analysis. Nonlinear regression of CM dose-response viability curves in MKN-45, DLD-1, Caco2, CT26 and L929 cells after treatment with CM at 0-100 µg/mL. IC₅₀ values were calculated by nonlinear regression.

Fig. S7. Intracellular GSH depletion induced by CM. Quantification of intracellular GSH levels in CT26 cells after treatment with CM at the indicated concentrations for 1, 6, 12 and 24 h. Data show time- and concentration-dependent depletion of cellular GSH after CM treatment. Data are presented as mean ± SD. Statistical significance was assessed by one-way ANOVA. * *p* < 0.05, ** *p* < 0.01, *** *p* < 0.001, **** *p* < 0.0001.

Fig. S8. Uncropped western blot membrane images. Original uncropped western blot images corresponding to the immunoblotting results shown in Fig. 5F, including FDX1, detergent-soluble DLAT, detergent-insoluble DLAT and their corresponding loading controls.

Fig. S9. Expanded tolerability assessment for intraperitoneal CM. Kaplan-Meier survival curves of mice over 7 days following intraperitoneal administration of CM at graded doses (0-60 mg/kg; n = 3 per group). Curves for 0-20 mg/kg overlap because no deaths occurred within 7 days.

Fig. S10. Histopathological safety evaluation of major organs. Representative H&E staining of heart, liver, spleen, lung, and kidney from mice treated with CM at the 20mg/kg dose, showing no overt histopathological damage, inflammatory infiltration, or tissue necrosis. Scale bars: 60 µm.

Fig. S11. Gross images of peritoneal lesions. Images documenting peritoneal tumor nodules and ascites burden across three groups at endpoint (yellow arrows indicate peritoneal metastatic nodules). Scale bar: 5 mm.

Fig. S12. Flow cytometry gating strategies used in this study. (A) Representative gating strategy for FITC-CM cellular uptake analysis in CT26 cells. (B) Representative gating strategy for JC-10-based mitochondrial membrane potential analysis. JC-10 red and green fluorescence signals were used to evaluate mitochondrial depolarization after CM treatment. (C) Representative gating strategy for immune-cell analysis of tumor and peritoneal samples. Single-cell events were sequentially gated before identifying T-cell subsets used for quantification of CD8⁺ T cells and CD4⁺Foxp3⁺ regulatory T cells.

Fig. S13. Flow cytometry comparisons of T-cell subsets. (A) Flow cytometry quantification of peritoneal CD8⁺ T-cell frequencies across groups. (B) Flow cytometry quantification of tumor-infiltrating CD4⁺Foxp3⁺ Tregs across groups. Data are presented as mean ± SD. Statistical significance was assessed by one-way ANOVA. * *p* < 0.05, ** *p* < 0.01, *** *p* < 0.001, **** *p* < 0.0001.
